# Plane Double-Layer Structure of AC@S Cathode Improves Electrochemical Performance for Lithium-Sulfur Battery

**DOI:** 10.3389/fchem.2018.00447

**Published:** 2018-10-29

**Authors:** Zengren Tao, Zhiyun Yang, Yafang Guo, Yaping Zeng, Jianrong Xiao

**Affiliations:** ^1^College of Science, Guilin University of Technology, Guilin, China; ^2^Guangxi Key Laboratory of Electrochemical and Magnetochemical Functional Materials, Guilin University of Technology, Guilin, China

**Keywords:** plane double-layer structure, active material, diffusion, polysulfide adsorption, lithium-sulfur battery

## Abstract

Due to the high theoretical specific capacity of lithium-sulfur batteries, it is considered the most promising electrochemical energy storage device for the next generation. However, the development of lithium-sulfur battery has been restricted by its low cycle efficiency and low capacity. We present a Plane double-layer structure of AC@S cathode to improve the electrochemical performance of lithium-sulfur batteries. The battery with this cathode showed good electrochemical performance. The initial discharge capacity of the battery with the structure of AC@S cathode could reach 1,166 mAhg^−1^ at 0.1 C. After 200 cycles, it still remains a reversible capacity of 793 mAh g^−1^ with a low fading rate of 0.16% per cycle. Furthermore, the batteries could hold a discharge capacity of 620 mAh g^−1^ after 200 cycles at a typical 0.5 C rate. The improvement of electrochemical performance is attributed to that the polysulfide produced during charge/discharge can be better concentrated in the cathode by the planar double-layer structure, thus reducing the loss of sulfur.

## Introduction

With the development of the global electric automobile and the requirement of users for the endurance mileage, the demand for high-energy density batteries has increased unprecedented (Manthiram et al., 2015). Lithium-sulfur batteries have a high theoretical specific capacity (1,675 mAh g^−1^), abundant reserves of sulfur and low production cost, it has been considered as the most promising electrochemical energy storage device (Song et al., 2016). As a result, many researchers have been attracted to improve electrochemical performance of Lithium - sulfur batteries.

However, there still are some problems restricting the commercialization of lithium-sulfur batteries:(1) Poor conductivity of sulfur (5 × 10^−30^ S cm^−1^) and discharge products lithium sulfides (3.6 × 10^−7^ S cm^−1^), poor reversibility of the discharge product lithium sulfides, easy to lose electrochemical activity, which would results in the loss of active-material. (2) During discharge, sulfur is first reduced to long chain polysulfide ions and dissolved into organic electrolytes. The dissolved long chain polysulfide ions ${\text{S}}_{\text{n}}^{2-}$ (*n* ≥ 4) migrate through the separator to the negative electrode and are reduced to short chain polysulfide ions. Among them, some short-chain polysulfide ions remigrate back to the positive electrode, resulting in a “shuttle effect.” The stronger “shuttle effect,” the more overcharge obviously of the battery. Another part of the short-chain polysulfide is further reduced to insoluble substance Li_2_S_2_/Li_2_S on the anode of lithium, results in a slow discharge/charge progress and a low practical capacity (Lin et al., 2017). In the course of repeated shuttles, continuous loss of active substances S, leads to the continuous attenuation of the battery capacity and the deterioration of the cycle performance. (3) The density of elemental sulfur (2.03 g cm^−3^) and Li_2_S (1.67 g cm^−3^) are different, obvious volume expansion will occur during the cycling process, resulting in the destruction of the sulfur cathode (Rauh et al., 1979; Peled et al., 1989; Cheon et al., 2004; Mikhaylik and Akridge, 2004; Barchasz et al., 2012; Manthiram et al., 2015; Urbonaite et al., 2015; Song et al., 2016).

In order to solve these problems, people have made a lot of meaningful efforts, for example, designing of cathode material (Xiao et al., 2015a; Zhao et al., 2017), the modification of separator (Guo et al., 2017a,b, 2018), the protection of negative electrode and the improvement of electrolyte system (Yan et al., 2013; Chen et al., 2017). In these researches, the exploration of sulfur cathode materials is particularly outstanding. Among cathode materials, the most representative are sulfur/carbon composites (Schuster et al., 2012; Ma et al., 2014), sulfur/conductive polymer composites (Xiao et al., 2012; Zhang et al., 2012) and sulfur/oxide composites (Ma et al., 2015; Yuan et al., 2017). However, these cathode materials are only synthesized by various physical and chemical methods, seldom works are on cathode structural design of lithium-sulfur batteries. Therefore, researchers designed the structure for cathode material, and proposed various hierarchical structure cathode materials, for example:Coaxial carbon nanotube structure (Zhang et al., 2016, 2018; Li X. et al., 2017; Wang et al., 2017), spherical nanodelamination structure (Huang et al., 2017; Ni et al., 2017; Cheng et al., 2018), and plane hierarchical structure (Chung et al., 2016; He et al., 2016; Huang et al., 2017; Li G. et al., 2017; Ni et al., 2017; Zhao et al., 2018). The preparation process of the planar hierarchical structure is relatively simple, and the prepared battery has good electrochemical performance. The hierarchical structure exhibits a lot of excellent properties, firstly, it can protect the active-material between the interlayer from escaping, thus reducing the loss of the active material and improves the cycle stability (Zhang et al., 2016; Ni et al., 2017). Secondly, the hierarchical structure can provide an efficient conductive network for the active substances thus improve the conductivity, then improving the Coulomb efficiency (He et al., 2016; Li G. et al., 2017). Thirdly, we can change the thickness of each layer, for example, increase the thickness of the active material layer, reduce the thickness of the barrier layer, and therefore increase the capacity of the active material (Chung et al., 2016). Therefore, the electrochemical performance of lithium-sulfur battery cathode materials can be improved by using hierarchical structure cathode materials.

Activated carbon has porous structure and good adsorption performance, sulfur can be filled into the mesh of activated carbon and can be adsorbed strong, so the sulfur loss can be reduced by using activated carbon as the framework material of sulfur (Ji et al., 2009; Schuster et al., 2012; Lee et al., 2014; Zhang J. et al., 2014; Zhang S. et al., 2014; Xiao et al., 2015b). In this study, the sulfur and carbon planar double layer structure cathode materials were prepared by using activate carbon (AC) and sulfur in different proportions. We propose a simple preparation method to fabricated cathode materials. The test results show good electrochemical performance: The initial discharge specific capacity of a lithium-sulfur battery with two-layer structure of AC@S cathode could reach 1,166 mAh g^−1^ at 0.1 C. After 200 cycles, it can still deliver a reversible capacity of as high as 793 mAh g^−1^ with a low fading rate of 0.16% per cycle, and a capacity-retention rate of 68% after 200 cycles. The reason for the hierarchical cathodes display good cyclability is explored by analyzing their activation process and the excellent polysulfide retention brought about by the hierarchical electrode structure.

## Experimental section

### Preparation of AC/S active material

First, S and AC (In different proportions 9:1, 1:9, 8:2, 2:8, 7:3, 3:7) were first placed in an agate mortar and ground for 1 h before being transferred to a polytetrafluoroethylene reaction vessel. Then, to exclude the residual air so that S would not be oxidizing at a high temperature, we ensured that the reaction vessel was static and open in an argon-filled glove box for 0.5 h. After that, the reaction vessel removed from the glove box and heated at 155°C for 12 h. At this temperature, the melted sulfur can easily penetrate into the pores of AC. Finally, the AC/S composite was obtained after cooling down to room temperature.

### Preparation of hierarchical cathodes

The working electrodes were prepared from various ratios a mixture of the S/AC composite, Super P, and polyvinylidene fluoride (PVDF) binder in N-methyl-2- pyrrolidinone solution (NMP) with a mass ratio of 8:1:1. The mixed active-material paste was first dropped onto the middle of the aluminum-foil current collectors. The following five samples of double-layer structure cathode cells were prepared: The first layer was 8: 2, the second layer was 2:8 and marked AC/S-1; The first layer was 9: 1, the second layer was 1:9 and marked AC/S-2; The first layer was 7: 3, the second layer was 3:7 and labeled AC/S-3; The first layer was S, the second layer was AC and marked AC/S-4; The sulfur monolayer labeled AC/S-5. The first layer is about 14 μm, and the second is about 13 μm. These samples were dried in a vacuum oven at 60°C for 12 h before cutting. Finally, the cathode was punched into a disk measuring 14 mm in diameter for assembling.

### Cell assembly

The CR-2025-type button cells is using to tests all of the electrochemical for the sulfur cathode, which were assembled in an argon-filled glove box using various ratios a mixture of the S/AC composite plane double layer structure anode/separators and Li metal as the counter electrode. The electrolyte used in this study was 1 M Li TFSI/DME + DOL (1:1, v/v) containing LiNO3 (1 wt%). The hierarchical cathodes and lithium anodes were connected to, respectively, an aluminum tab and a nickel tab. Pouch cells were sealed with an aluminum soft packaging film. The assembled coin and pouch cells were allowed to rest for 30 min at 25°C before electrochemical measurements.

### Material characterization

Field emission scanning electron microscopy (SEM, HITACHIS-4800) was used to characterize the morphology at before and after 200 cycles of the AC/S-1 cathode. The distribution of the elements on the surface of the AC/S-1 cathode was identified with an energy dispersive spectrometer (EDS). X-ray diffraction (XRD, X'Pert PRO) was used to characterize the crystallinity of AC/S-X (*X* = 1, 2, 3, 4, 5).

### Electrochemical measurement

The CHI750E electrochemical workstation was used to measure the cyclic voltammetry (CV) and electrochemical impedance spectroscopy (EIS). Discharge and charge profiles and cyclability data were evaluated under galvanostatic conditions between 1.5 and 2.8 V with a programmable battery cycler. CV measurements were performed at a scan rate of 0.01 mV s^−1^ in the voltage range of 1.5–3.0 V. The EIS tests were carried out at the frequency range of 0.01–100 kHz with a perturbation amplitude of 5 mV.

## Results and discussion

### Configuration and morphology

**Figure 1:**
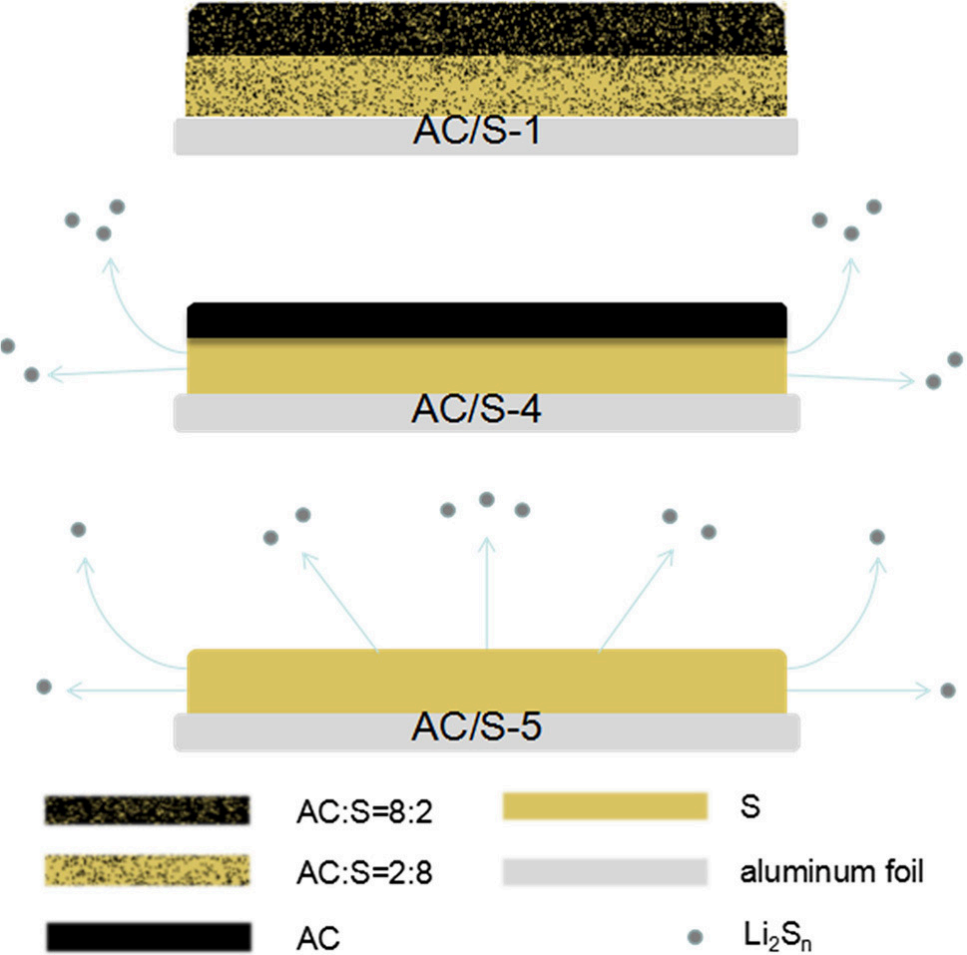
Electrode configuration of the hierarchical cathodes with AC/S-X(X = 1, 4, 5) cathode.

The configuration of the hierarchical cathode is shown in Figure 1. In the plane-double layer structure, the electrochemically stable carbon film is covered onto a layer of active-material coating as a carbon-film shield to form the hierarchical cathode. Polysulfides Li_2_S_n_ (4≤n≤8) of AC/S-5 monolayer cathode can be easily escaped from the cathode without protection, resulting in a great loss of S. Although the AC/S-4 cathode is protected by carbon layer, the polysulfide can easily escape from the edge of cathode and cause sulfur loss. However, due to the existence of AC in both layers of the cathode of AC/S-1 plane double-layer structure, because sulfur coated by activated carbon of each layer, the polysulfide produced during discharge can be adsorbed by AC and migrated to the upper layer, which can provide enough space for polysulfide, thus, the loss of sulfur can be minimized.

**Figure 2:**
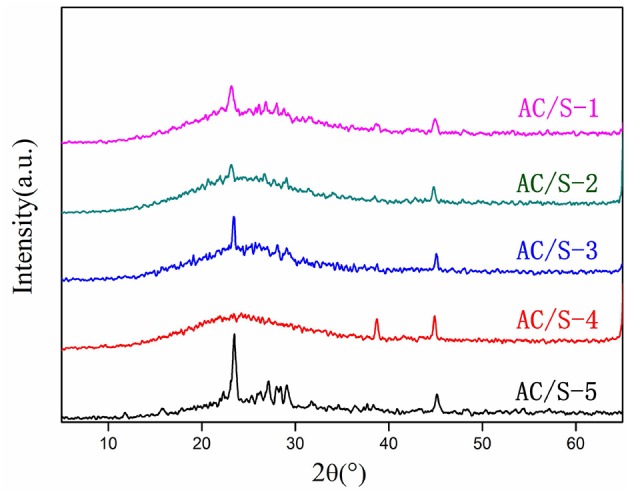
The XRD patterns of AC/S-X.

From Figure 2. The single mass sulfur peak is strong and sharp (AC/S-5), and there are obvious diffraction peaks in the whole scanning range, indicating that the material has stronger crystal structure, and the main diffraction peak is located at 2θ = 23.083° and 2θ = 27.769°, which belongs to the skew square type diffraction peak and is a typical S8 structure (Yuan et al., 2009; Xiao et al., 2015b). The activated carbon had a “steamed bread peak” at 2θ = 20~30°, which showed an amorphous state. The four kinds of cathode materials showed typical “steamed bread peak” with activated carbon, and there were diffraction peaks of crystal sulfur, but the intensity of the crystal peak of sulfur decreased obviously. The results show that both crystallinity and amorphous states of sublimated sulfur were found in the composites. It can be seen that the diffraction peaks of AC/S-1 and AC/S-3 cathode are obvious because the high content of S in the surface layer (the content of S of AC/S-1 is 20% and the content of S of AC/S-3 is 30%), and most of the sulfur goes into the nano-pores of activated carbon, but a small amount of sulfur is deposited on the surface of carbon material. However, the diffraction peaks of AC/S-3 and AC/S-4 cathode materials are weakened, which is due to the low content of S in the surface layer (the content of S of AC/S-3 is 10% and the content of S of AC/S-3 almost 0), so basically all the S enter into the nano-micropores of activated carbon, and enhance the amorphous structure of the materials.

**Figure 3:**
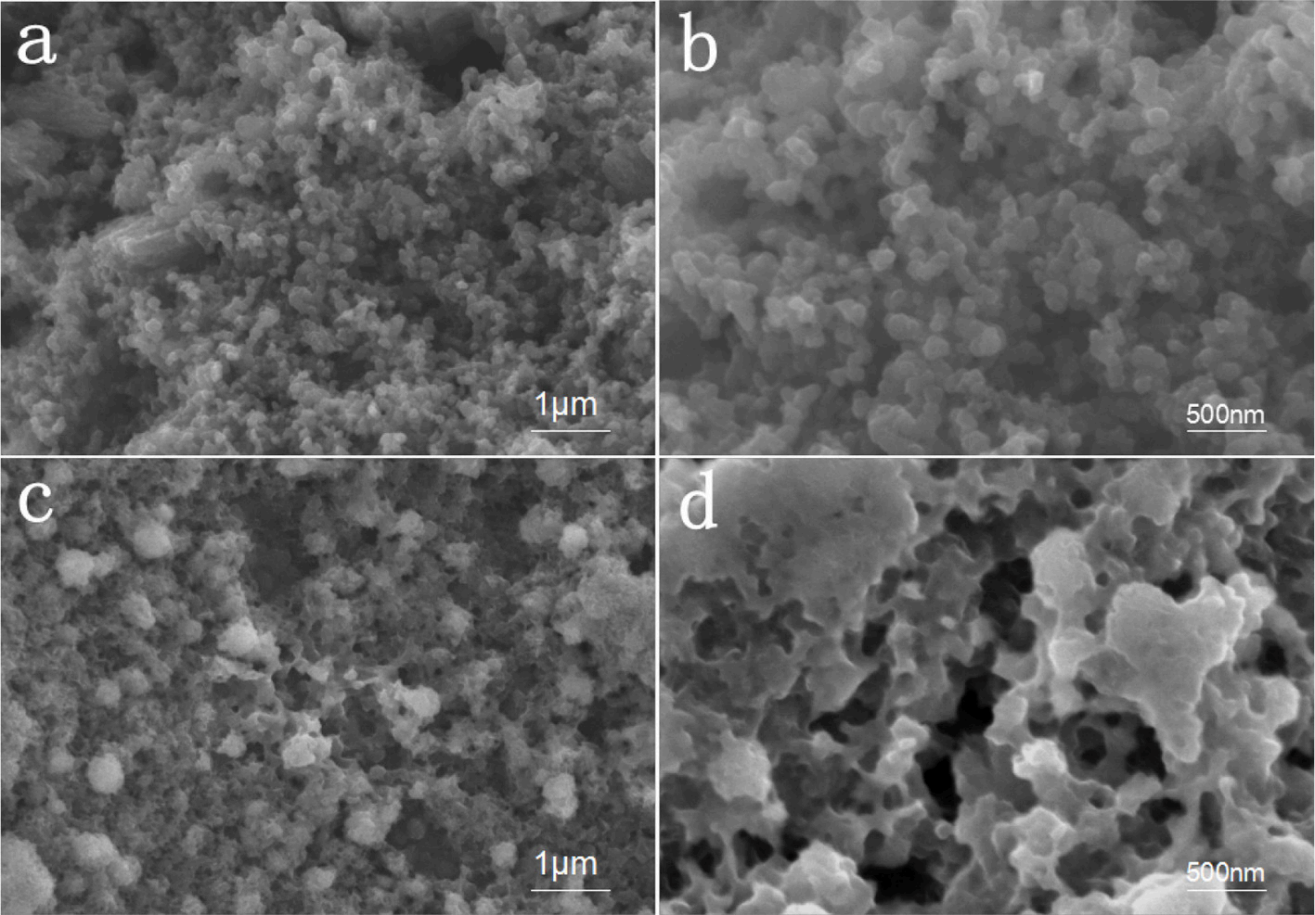
High-magnification SEM images of the AC/S-1 cathode **(a,b)** before cycling and **(c,d)** after 200 cycles at 0.1 C.

The SEM diagram of the positive pole of AC/S-1 before and after 200 cycles is shown in Figure 3. Before cycling, the surface of the AC/S-1 cathode is covered with activated carbon particles of varying sizes bonded together by PVDF, and there are abundant pores between the particles, these pores can not only store lithium-sulfur battery electrolyte, but also provide space for intermediate products and inhibit the shuttle effect during reaction.

**Figure 4:**
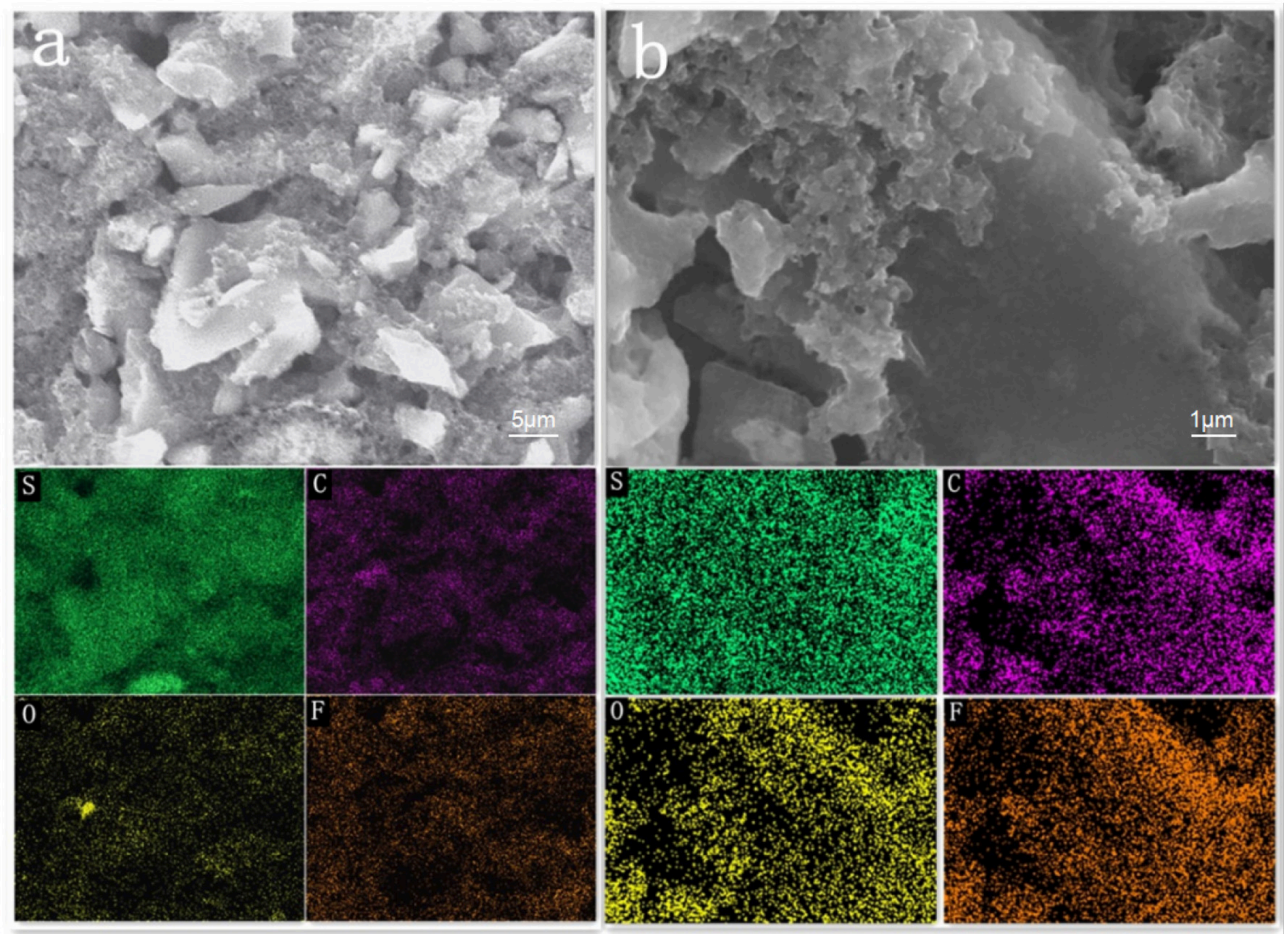
Low-magnification SEM images and elemental mapping of the AC/S-1 cathode **(a)** before and **(b)** after 200 cycles at 0.1C.

The SEM contrast diagram of AC/S-1 cathode after 200 cycles at 0.1 C is given in Figures 3c,d. It is clear from the diagram that the surface of the carbon particles at the positive pole is relatively smooth before cycle. The surface of the carbon particles deposited has a large amount of scale-like pimples after cycle, these uniformly distributed pimples are intermediate products Li_2_S_n_, which is intercepts by the AC/S-1 cathode during the charging and discharging of the battery (Guo et al., 2017a,b). The EDS surface scan of the four elements (S, C, O, F) of AC/S-1 cathode before and after cycle in Figure 4a. The distribution of element C is uneven, and there is obvious agglomeration, but the signal of three elements O, F, S is very weak. After cycles, the signal of element C was strengthened in Figure 4b, and the phenomenon of agglomeration disappeared, while the signals of three elements of O, F, C were obviously enhanced and uniformly dispersed. These differences can be attributed to the adhesion of polysulfide to active carbon hierarchical cathode material and the accumulation of electrolyte.

**Figure 5:**
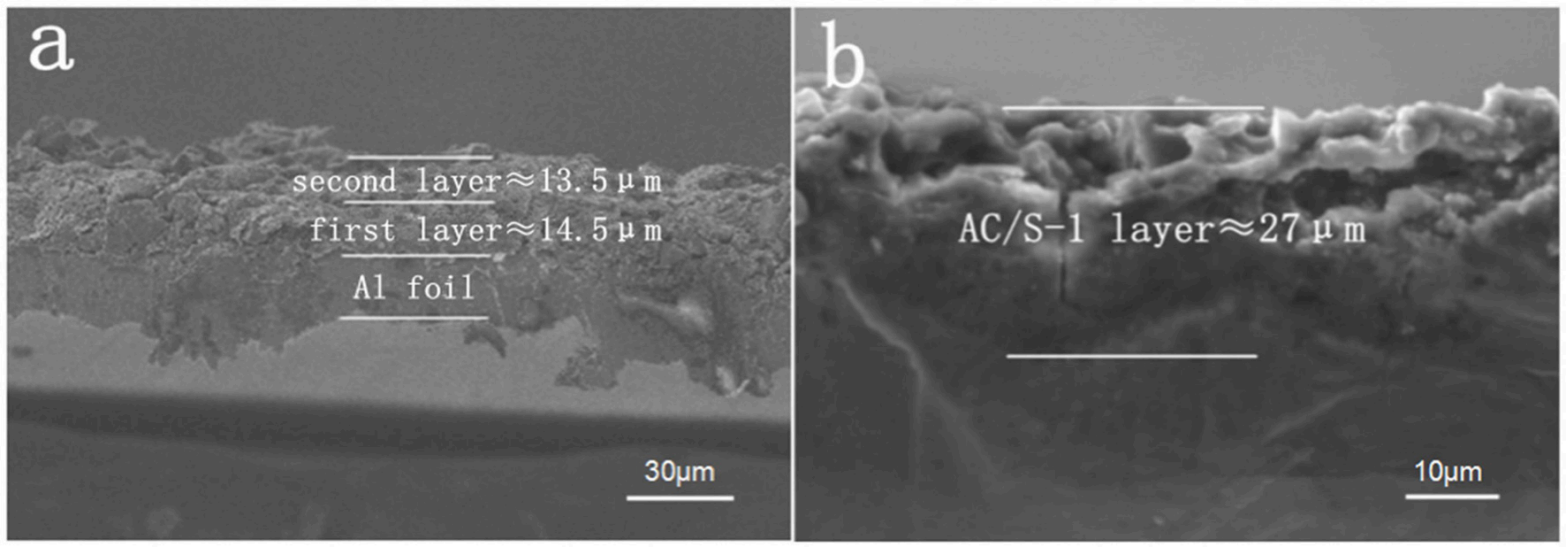
SEM images of the AC/S-1 cathode cross-section of **(a)** before cycling and **(b)** after 200 cycles at 0.1 C.

The contrast diagram of SEM cross section of AC/S-1 cathode before and after cycle are shown in Figures 5a,b. It is obvious that the delamination can be seen in the hierarchical structure cathode before the cycle, but it difficult to distinguish after 200 cycles at 0.1 C. This is because of the polysulfide continuous diffusion to the surface layer, and the active substance uniform diffusion throughout the cathode during the battery cycle.

**Figure 6:**
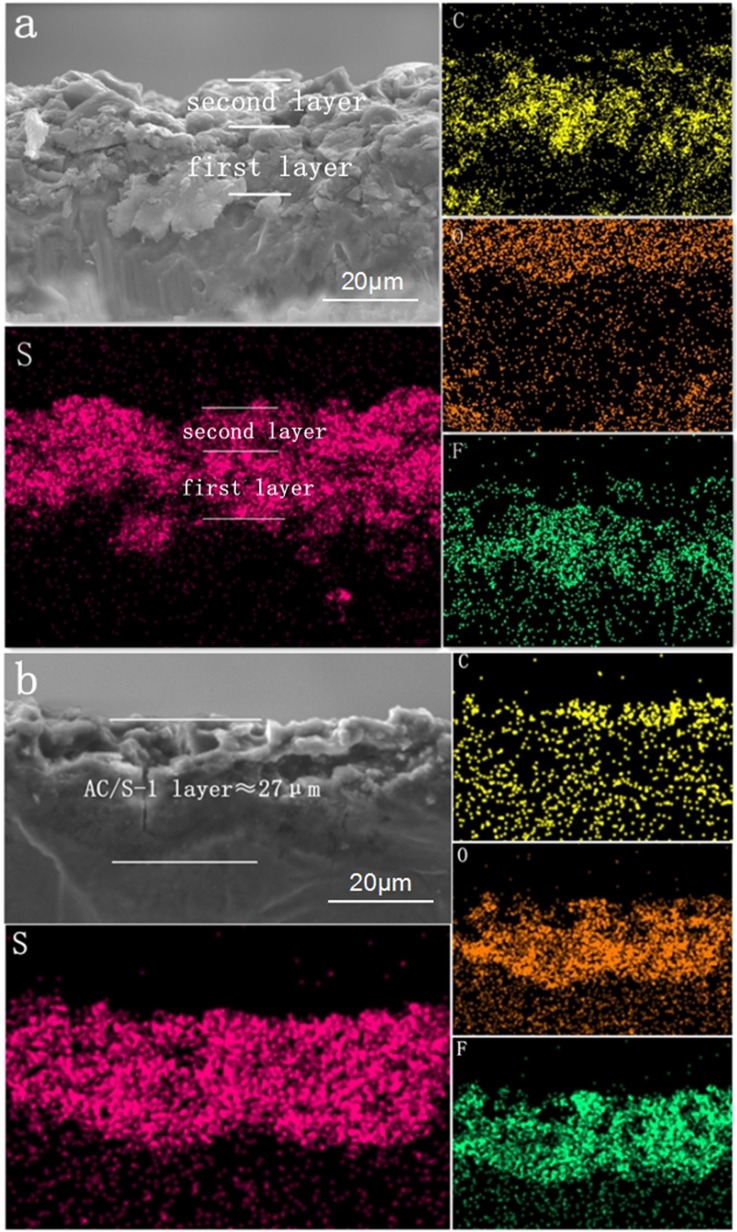
SEM images and elemental mapping of the AC/S-1 cathode cross-section of **(a)** before cycling and **(b)** after 200 cycles at 0.1 C.

The cross section of the layered cathode by EDS are presented in Figures 6a,b. It can be seen from the diagram that the content of S in the first and second layers is obviously different before the cycle. It is evident that sulfur-containing species start to diffuse out from the active-material layers and are absorbed by AC of upper layer, as a result of the stabilized polysulfide migration (Chung et al., 2016). After 200 cycles, the diffusing polysulfidesare stabilized within the hierarchical electrode and the elemental sulfur signals are, therefore, uniformly distributed in the conductive matrix.

### Electrochemical stability

**Figure 7:**
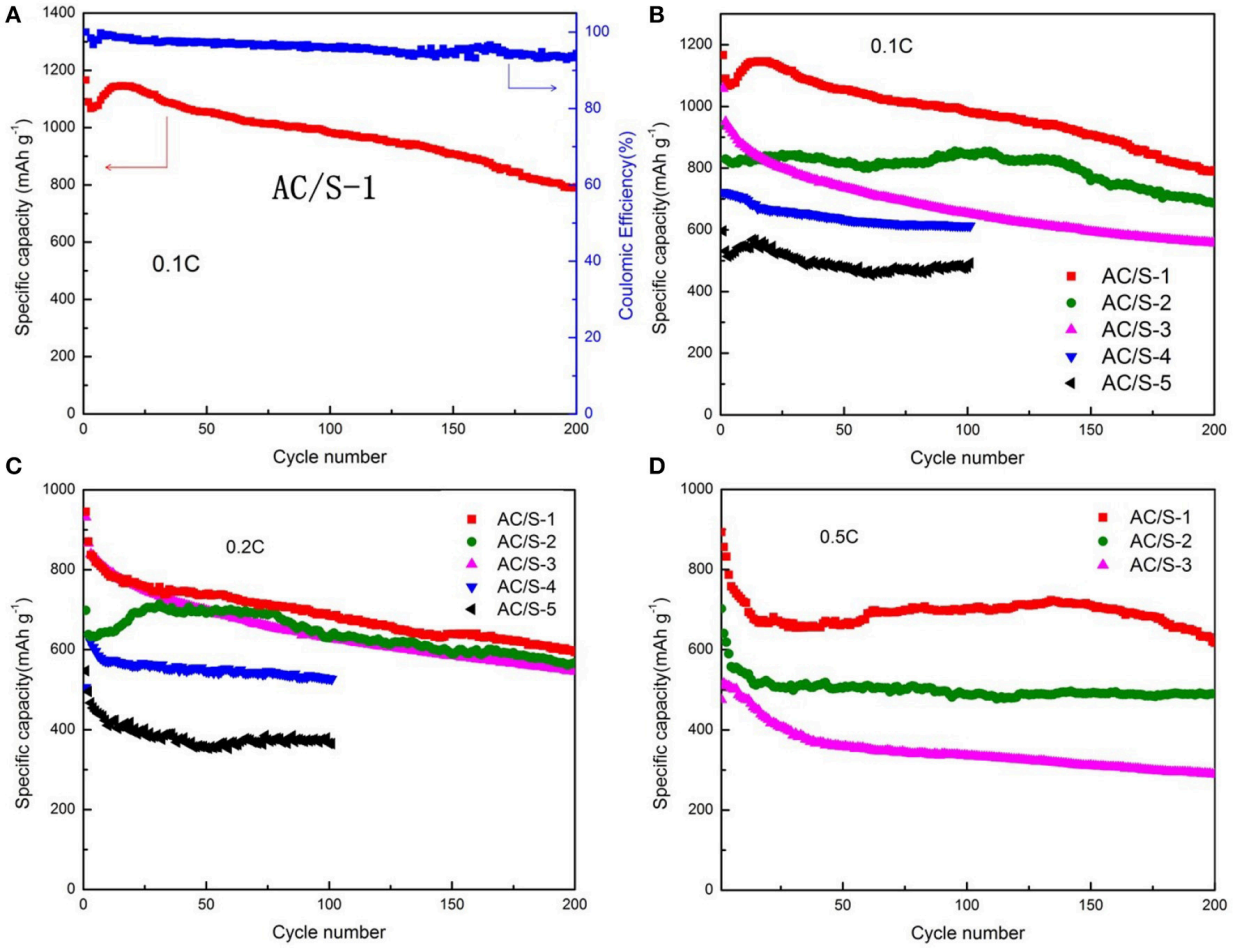
Cycle performance of Li-S cells with AC/S-X cathode at **(A)** 0.1 C, **(B)** 0.1 C, **(C)** 0.2 C, **(D)** 0.5 C.

In order to determine what proportion of AC and S for prepare the planar double - layer structure cathode play the best role, and greatly improve electrochemical performance of lithium-sulfur battery. We have measured the cycle performance and the C-rate performance of the five kinds of AC/S-X(*X* = 1, 2, 3, 4, 5) batteries, at different discharge current rates. Excellent electrochemical utilization and stability allow the hierarchical cathodes to attain high discharge capacity and long-term cyclability for 200 cycles at various cycling rates as shown in Figure 7. Coulomb efficiency of AC/S-1 greater than 90% (Figure 7A), it indicate that the structure cathode battery has higher reversible capacity. As can be seen from the Figure 7B, the initial discharge capacities of AC/S-X(*X* = 1, 2, 3, 4)at 0.1 C reached 1,166,1,057,939,721 mAh g^−1^,AC/S-X(*X* = 1, 2, 3)cells at after 200 cycles, the reversible discharge capacities were 793,686,556 mAh g^−1^,AC/S-4 cells at after 100 cycles is 612 mAh g^−1^ respectively. Compared with the original sulfur monolayer structure cell (AC/S-5), the cycle performance obviously improved, especially when the first layer AC: *S* = 2: 8 and the second layer AC: *S* = 8:2, the performance tends to be optimal. This is because when the AC content of first layer reaches 20%, it can provide enough pores to adsorb S, when the AC content of second layer reaches 80%, it can effectively prevent the polysulfide from escaping in the cathode, and as far as possible to reduce the loss of S, alleviates the occurrence of the shuttle effect. Therefore, the first discharge specific capacity of planar double-layer structure battery with AC/S-1 ratio can reach to 893 mAh g^−1^ at 0.5 C, after 200 cycles the battery still maintain a good capacity(about 620 mAh g^−1^), as shown in the Figure 7D.

**Figure 8:**
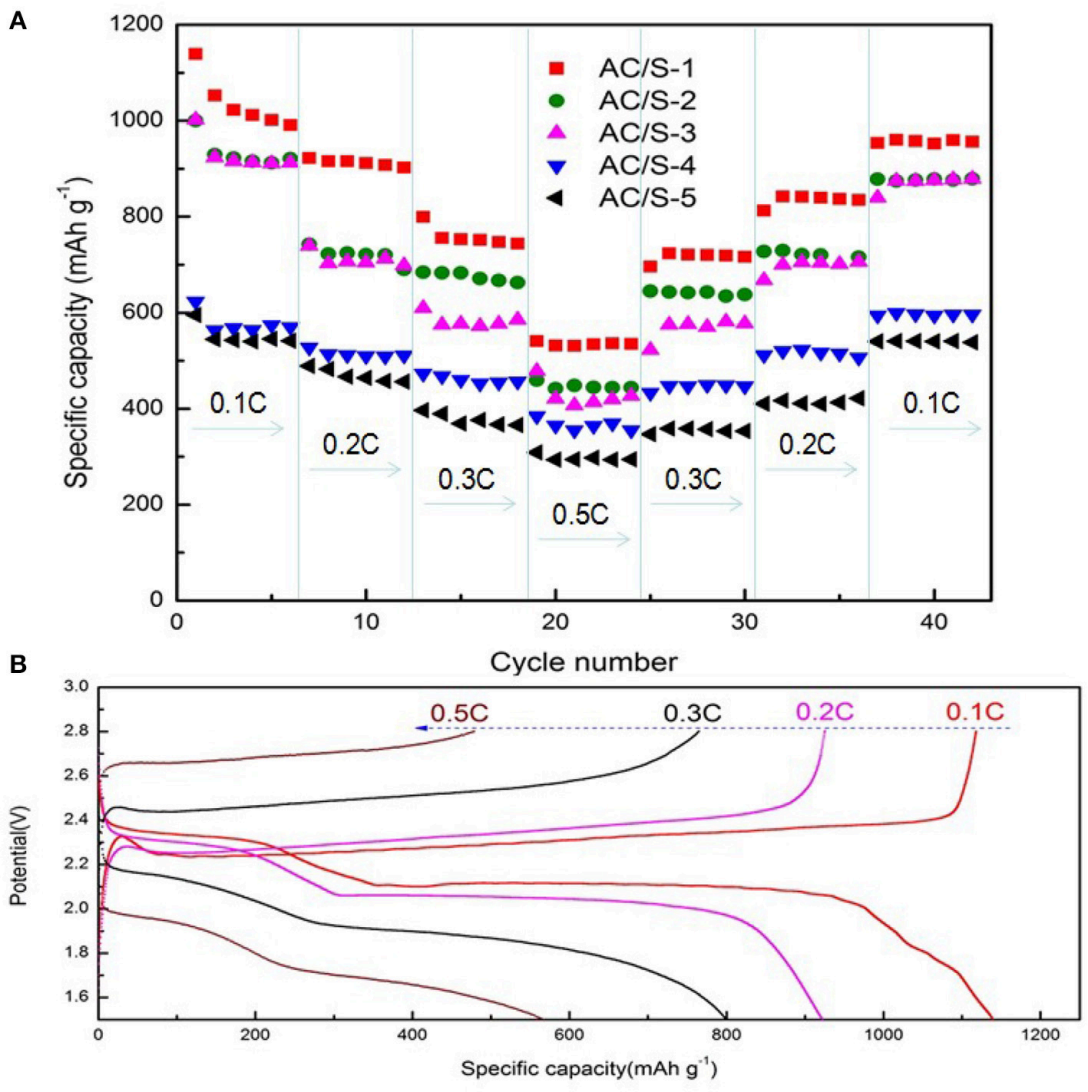
**(A)** Rate performance of AC/S-X, **(B)** the initial discharge/charge curves of the AC/S-1 cathode at various C rates from 0.1 C to 0.5 C.

Figure 8A shows the C-rate performance of the battery, which is another important aspect to evaluating the electrochemical performance of lithium sulfur battery. The AC/S-X batteries was tested at varying rates (0.1 C → 0.2 C → 0.3 C → 0.5 C → 0.1 C). It can be seen from the graph, that the initial discharge capacity of the cell with the AC/S-1 hierarchical cathode reached 1139 mAh g^−1^. Although the capacity of the battery declined rapidly in the first six cycles, the capacity of the battery began to stabilize with the increase of the current ratio. Capacity of the battery slow descent from 990 mAh g^−1^ at 0.1 C to 922, 799, and 540 mAh g^−1^ at 0.2, 0.3, and 0.5 C respectively. More importantly, when the current rate is back to 0.1 C, the capacity of the AC/S-1 planar double-layer structure battery is obviously higher than that of the other four sample batteries, it is shown that the planar double layer structure of this AC/S ratio has a more effective limiting effect on the diffusion of polysulfide. From the charge/discharge profiles at various rates shown in Figure 8B, the AC/S-1 planar double-layer structure characteristic can be clearly identified at 0.1 C, has a longer charge/discharge curve, indicative of low polarization, sulfur can be fully react to form Li2S2/Li2S, thus enhances the utilization of active material (Zhang et al., 2017).

**Figure 9:**
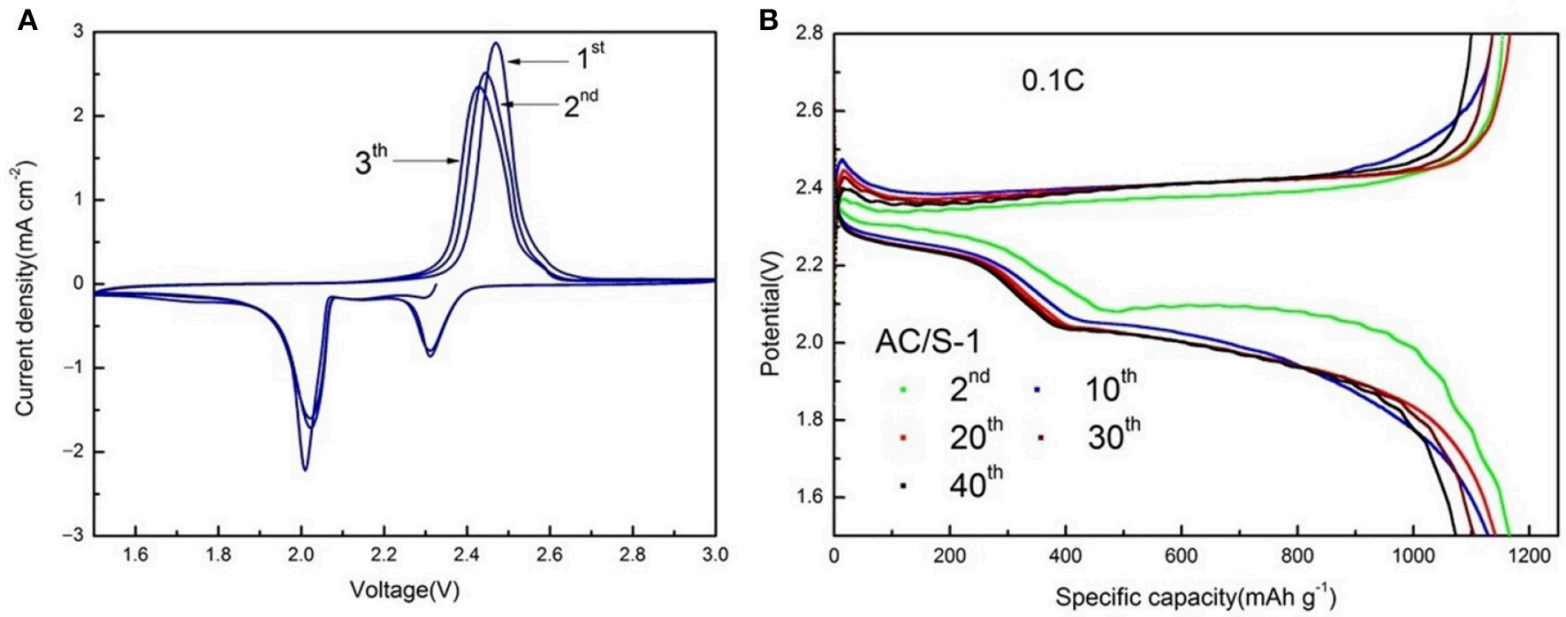
**(A)** CV curves at the scan rate of 0.1 mV s^−1^ and **(B)** from 2nd to 40th discharge/charge curves of AC/S-1 cathode at 0.1 C between 1.5 and 2.8 V.

Typical cyclic voltammograms (CVs) at a scan rate of 0.1 mV s^−1^ and the potential range of 1.5–3 V are presented in Figure 9A. There are two cathodic peaks, relating to the formation of high-order S_n_ (4≤n≤8) and insoluble Li_2_S_2_/Li_2_S. Therefore, in the subsequent anodic scan, two oxidation peaks are observed, corresponding to the oxidation of insoluble Li_2_S_2_/Li_2_S to soluble polysulfides and polysulfides to sulfur (Lee et al., 2002; Zhang, 2013; Zhu et al., 2016; Zhang et al., 2017). While comparing with the other batteries, the AC/S-1 planar double-layer structure battery possesses larger and stable current density of redox peaks in CVs, explaining a low polarization, good reversibility, and excellent cycle stability. Thus, the cathode of the planar double-layer structure can improve the electrochemical performance of the battery; in particular, the cathode of the AC/S-1 ratio has the greatest influence on the improvement of the electrochemical performance. Here are the charge-discharge curves of AC/S-1 batteries at the 2nd, 10th, 20th, 30th, and 40th times, at 0.1 C are presented in Figure 9B. The upper-discharge plateau at 2.3 V indicates the reduction of sulfur leads to the formation of soluble polysulfide. The fast sulfur(solid)-polysulfides(liquid) reduction reactions involve the formation, dissolution, and diffusion of polysulfides (Huang et al., 2015; Li G. et al., 2017).

**Figure 10:**
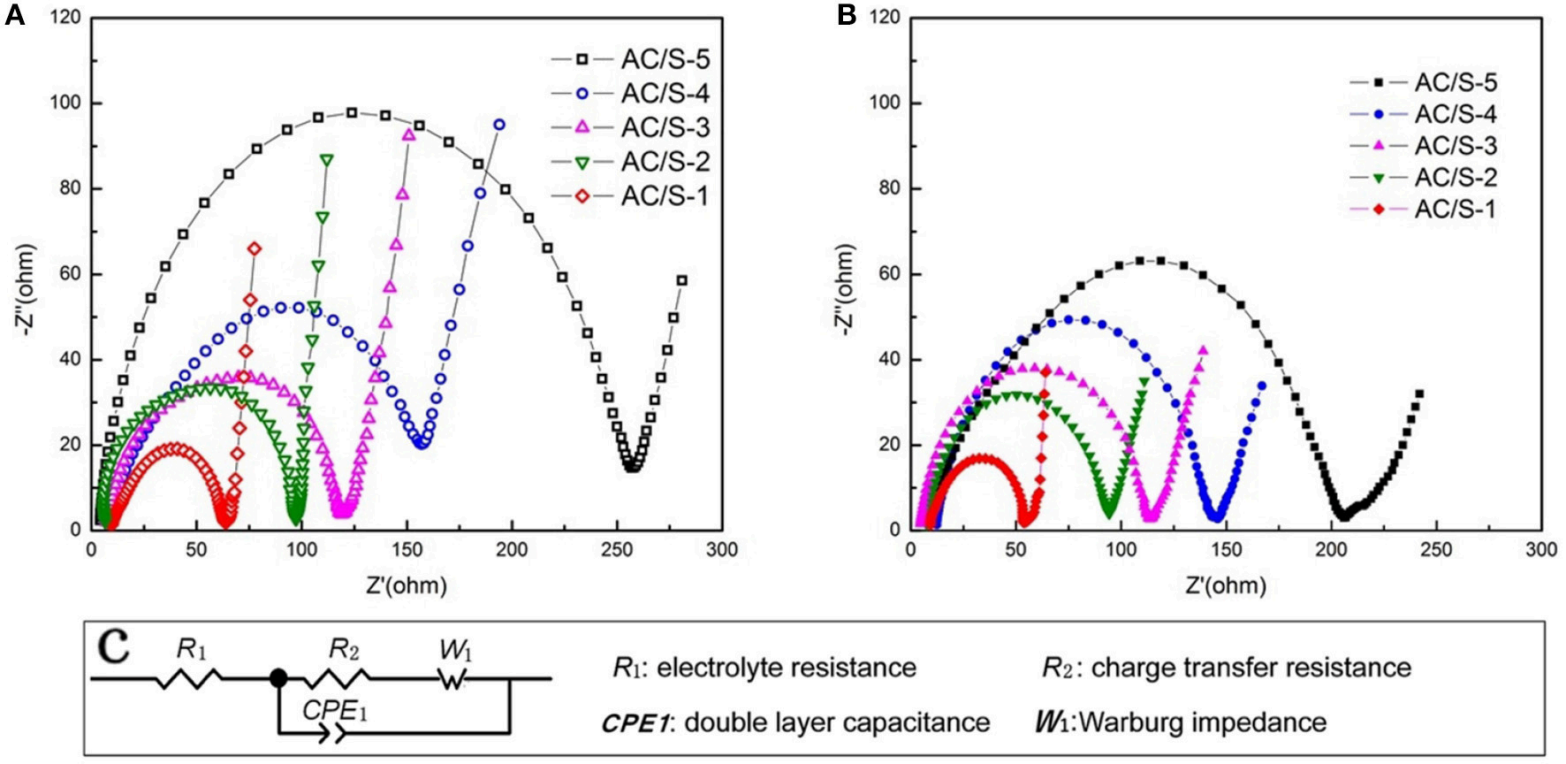
Electrochemical impedance spectrum (EIS) of the cells with AC/S-X cathode **(A)** before cycling and **(B)** after 200 cycles at 0.1 C, **(C)** Equivalent circuit used for fitting the impedance spectra.

To get a further understanding with the contribution of the designed plane hierarchical structure on the performance, electrochemical impedance spectra (EIS) is shown in Figure 10. The electrochemical workstation was used to test the electrochemicl impedance with the frequency range of 0.01 Hz-100 kHz. The R1 in the illustrations denotes the resistance of the electrolyte, and *R*_2_ denotes the charge transfer resistance of the battery, *W*_1_ indicates that the Walburg diffusion impedance, *CPE*_1_ is a constant phase original. Before and after the cycle, the electrochemical impedance curves of the sample cells are composed of the medium and high frequency band semicircle (corresponding to the charge transfer resistance) and low frequency band sloping line (corresponding to the Warburg impedance). In addition, the ohmic resistance of the lithium-sulfur battery is derived from the intercept between the coordinate axis and the curve in the high frequency range (Kolosnitsyn et al., 2011; Deng et al., 2013; Zhang et al., 2015). By comparing the diameters of semicircle we know that the charge transfer resistance of the battery decreases before and after the cycle. Furthermore, due to the active-material redispersing process in AC/S-1 planar-double layer structure, enable the active-material to be fully utilized. The charge transfer resistance of the battery is the smallest after cycle.

## Conclusions

In summary, the planar double-layer cathode can enhance the electrochemical stability of the lithium-sulfur battery. The capacity attenuation rate of the battery is 0.16% and long term cyclability (more than 200 times) still keeps high capacity. The planar double-layer cathode used as the charge/discharge platform has the potential to increase the sulfur content and applied to the battery. The migration processes of polysulfides were discussed by observation of the cross-section microstructure and elemental analysis. We attributed the more stable electrochemical performances to the special planar double-layer cathode, when active material moved to the upper layer, the structure would restrain the dissolve of polysulfides by the physical adsorption ability of activated carbon. Therefore, the utilization of the planar double-layer structure as sulfur cathodes presents a potential opportunity to ameliorate the long-term cycle stability of the corresponding Li-S batteries.

## Author contributions

ZT prepared all materials and performed electrochemical characterizations. ZY conducted SEM and XRD experiments. YG and ZT analyzed the data. ZT and YZ wrote the manuscript. JX supervised the implementation of the project.

### Conflict of interest statement

The authors declare that the research was conducted in the absence of any commercial or financial relationships that could be construed as a potential conflict of interest.
